# Ecological Status of Algeciras Bay, in a Highly Anthropised Area in South-West Europe, through Metal Assessment—Part I: Abiotic Samples

**DOI:** 10.3390/toxics12030163

**Published:** 2024-02-20

**Authors:** María José Casanueva-Marenco, María Dolores Galindo-Riaño, María Dolores Granado-Castro, Margarita Díaz-de-Alba

**Affiliations:** Department of Analytical Chemistry, Institute of Biomolecules (INBIO), Faculty of Sciences, International Campus of Excellence of the Sea (CEI-MAR), University of Cadiz, Campus Rio San Pedro, 11510 Puerto Real, Cadiz, Spain; mariajose.casanueva@uca.es (M.J.C.-M.); dolores.granado@uca.es (M.D.G.-C.); margarita.diaz@uca.es (M.D.-d.-A.)

**Keywords:** metal bioaccumulation, metal pollution, fractionation, pollution indexes, water, sediment

## Abstract

The ecological status of Algeciras Bay (South-west Europe), highly influenced by anthropogenic activities, was assessed by monitoring Zn, Cd, Pb, and Cu in water and sediment samples. Total contents and metal fractions with different availabilities and their spatial-seasonal distribution were determined. The trend in water and sediment contents were Zn > Pb ≈ Cu > Cd, without significant seasonal variations. Sites 3 and 4, closest to industrial activities, had the highest metal concentrations, mainly in sediments. Cd showed low partition coefficient in water, indicating higher bioavailability. Total metal content in sediments exceeded the threshold effect level for Cu and were close to Pb. The BCR procedure revealed the highest availabilities for Cd and Pb, due to its higher content in exchangeable and reducible fractions. Higher Pb levels (21.4 ± 5.1 mg/kg) were found in sediments of this bay compared with other ecosystems. Pollution indexes for sediment quality revealed that site 3 was the most polluted (CF = 7.12 and I_geo_ = 2.25). For an integrative study of the ecological status of this significant bay, these results have been complemented with the metal evaluation in benthic and benthopelagic fish tissues in *Ecological status of Algeciras Bay, in a highly anthropised area in south-west Europe, through metal assessment*—*Part II: Biotic samples*.

## 1. Introduction

Aquatic ecosystems have been subject to strong anthropogenic influence due to population and industrial growth. Among pollutants, metals have become a serious threat to the environment and human health due to their non-degradable nature, toxicity, and bioaccumulation [1,2]. Total metal content is often a poor measure of the bioavailability, mobility, or toxicity of heavy metals in aquatic ecosystems, as these contaminants exhibit different physical and chemical behaviours depending on their chemical forms [3,4].

Trace metals in water occur in different forms, which are usually first classified in an operational way into particulate (i.e., metal ions adsorbed on various solid and colloidal particles, precipitated, neutral ionic pairs, etc.) and dissolved forms (i.e., simple inorganic species, organic, labile, and inert complexes, etc.) with different bioavailabilities. These forms affect the biogeochemical processes that involve trace metals in ecosystems, such as the degree of adsorption to suspended matter, the deposition on sediments, the migration rate to sediments, and overall transport within aquatic ecosystems, among others [5,6,7]. When heavy metals are released and incorporated into aquatic systems, they tend to bind to particulate matter and can eventually become part of bottom sediments that act as sinks. Thus, sediments play an essential role in receiving and releasing these and other compounds from and to the water column, where physical, chemical, and biological processes are involved [8,9,10].

Trace metals interact with the sediment matrix through different binding mechanisms, including adsorption to mineral surfaces, association with carbonates, Fe/Mn oxyhydroxides, organic matter, sulphides, and the lattice of refractory crystalline minerals, such as silicates. The accumulation and mobility of heavy metals in sediments is dependent on and is controlled by several factors, such as the nature of the sediment particles, the properties of the adsorbed compounds, and the prevailing physico-chemical conditions [11,12]. In this context, it is important to note that the bioaccumulation of metals in biota from sediment is not directly related to the total metal concentration, but rather to more bioavailable forms of metals, such as exchangeable, reducible, or oxidisable [13]. Since heavy metals exhibit different environmental behaviours depending on their chemical forms and/or interactions, the geochemical fractionation of trace metals in sediments to assess their potential mobility, bioavailability, and toxicity to aquatic ecosystems has gained greater importance in recent decades. For this purpose, metal fractionation using sequential extraction methods has been widely used in order to differentiate metal forms by simulating the mobilisation and retention of these species in the natural environment using changes in environmental conditions such as pH, redox potential, and degradation of organic matter. These fractionation procedures are based on the selection of different reagents with increasing dissolving power in relation to the geochemical phases [14,15,16,17,18].

The monitoring studies carried out in this bay focused on the metals zinc (Zn), cadmium (Cd), lead (Pb), and copper (Cu). Pb and Cd are considered non-essential metals and are included in the EU-WFD list of priority hazardous substances, while Zn and Cu are essential for living beings and act as important cofactors in many biochemical processes. However, both metals may become toxic above a threshold concentration [19,20]. The presence of industries, settlements, and ports in this area can increase the levels of these metals and pose a threat to both aquatic life and human health. In this area, the main sources of Zn include fossil fuel burning, traffic emissions (gasoline), or industrial/domestic wastewater; Cd sources include metal smelting and refining, fuel burning, metal processing, and wastewater treatment facilities; Pb sources are petrochemical industries processes, coal combustion, traffic emissions (maritime transport), and ocean engineering; and Cu sources include traffic emissions (diesel oil), ocean engineering, and industrial/domestic wastewater. Phosphate fertilizers may contribute to high levels of Cd and Cu [21,22]. All these anthropogenic influences are also causing great social concern.

The geographical location of this bay between the Atlantic Ocean and the Mediterranean Sea, and between the European and African continents, is reflected in its great maritime importance and its use as a privileged port area. Although it has been environmentally studied, to date, there are no published studies on integrative research about water, sediment, and important fish species of this bay with the aim of identifying hazardous effects caused by Zn, Cd, Pb, and Cu concentrations on this ecosystem and also human health. In this *Part I: Abiotic samples* the main objectives were to assess the ecological status of water and sediments in this ecosystem by (i) determining the total levels of Zn, Cd, Pb, and Cu, and evaluating their availability in water and sediments; (ii) studying their spatial-seasonal distribution, as well as the relationships between the contents in solid–liquid phases to find the possible source of metals in this stressed bay; (iii) comparing the results with international guidelines and local regulations in order to classify the level of pollution of this aquatic ecosystem; (iv) comparing the results with values from other anthropogenically influenced ecosystems; and (v) evaluating different indexes of sediment quality pollution in order to estimate the enrichment level and ecological impact of these heavy metals. The data from this work have been taken into account in the study of benthic and benthopelagic fish species from the same sampling sites in *Ecological status of Algeciras Bay, in a highly anthropised area in south-west Europe, through metal assessment*—*Part II: Biotic samples*, as an integrative way to assess the ecological status of this significant bay.

## 2. Materials and Methods

### 2.1. Description of the Area and Sampling Sites

The Bay of Algeciras is an important industrialised area located on the Mediterranean coast of southwestern Spain limited by Punta del Carnero (Algeciras) and Punta Europa (Gibraltar) [23]. This bay covers an area of about 9 × 11 km^2^, with a maximum depth of almost 400 m [24]. Five cities with more than 275,000 inhabitants are located around the bay (Algeciras: 122,368, Los Barrios: 24,069, La Línea de la Concepción: 63,271, San Roque: 33,018) [25]; Gibraltar: 32,714; total: 275,440 [26]). The bay holds two important ports sited on Algeciras and Gibraltar with intense marine traffic that can cause discharges and accidental spills [27], and also numerous industrial plants distributed along its coastline [24] including stainless steel manufacturing plants, refineries, and petrochemical installations, thermal power plants, ironworks, shipyards, and docks [23,28,29]. Furthermore, urban wastewater discharges may occur due to the high population density of the bay, coming from the main population centres of the cities of Algeciras, Los Barrios, San Roque, La Línea de la Concepción, and Gibraltar. The bay also receives the water discharge from the Guadarranque and Palmones rivers. The water of the bay has a high turnover because of its proximity to the Strait of Gibraltar, where the Mediterranean Sea and the Atlantic Ocean meet with strong currents. These geographical conditions could disperse pollutants in the water [23]. Nevertheless, marine pollution is a realistic risk and a major problem in this area subject to persistent anthropogenic pollution.

**Figure 1:**
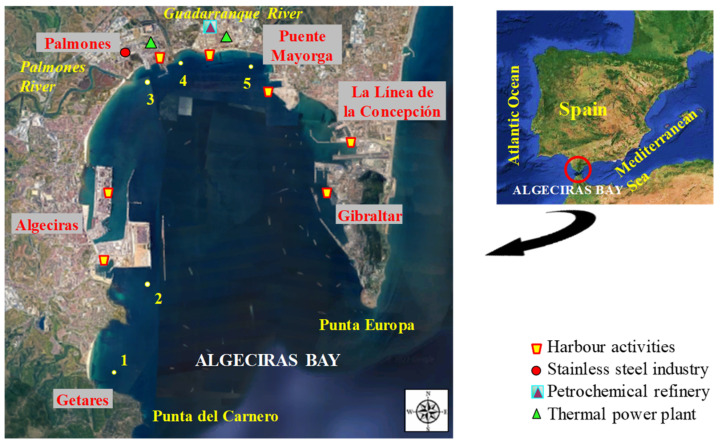
Map of Algeciras Bay showing the sampling sites and principal anthropogenic activities in the area.

Abiotic samples (water and sediment) were collected from five representative sampling sites (Figure 1): 1—*Getares beach* (control site with maritime traffic and limited urban influence), and four pollution hotspots named 2—*Isla Verde* (with road and maritime traffic due to the port activity of the Port of Algeciras), 3—*Palmones* (area characterised by the presence of a steel manufacturing plant, a thermal power plant, the Palmones river, and urban influence), 4—*Guadarranque* (close to a Chemical Pole with refineries and a thermal power plant, apart from the presence of the Guadarranque river and urban influence), and 5—*Puente Mayorga* (close to power thermal plants, port activities, and maritime traffic from the Port of Gibraltar).

The selection of these sites was based on previous studies [30], where the metal content in sediment samples from 17 sites along the Algeciras Bay was studied. The different samples were consecutively collected at four timepoints: sampling 1 (1st autumn), sampling 2 (1st spring), sampling 3 (2nd autumn), and sampling 4 (2nd spring). More information about sampling can be found in Table S1 of the Supplementary Material.

### 2.2. Equipment and Reagents

All analytical instruments and equipment used in this work are listed in Table S2 of the Supplementary Material.

All chemicals and standard solutions used for trace metal analyses were of Suprapur and Pro Analysis quality purchased from Merck (Darmstadt, Germany) or Sigma-Aldrich (Steinheim, Germany). The standard solutions required for the calibration curves were prepared by dilution of 1000 mg/L commercial standard solutions.

### 2.3. Collection, Pretreatment, and Analysis of Abiotic Samples

#### 2.3.1. Water

Water samples were taken directly from a boat at a depth of 0.3 m below the water surface, using a peristaltic pump (Cole-Parmer Instrument Co., Vernon Hills, IL, USA), rigid Teflon tubing, and flexible Tygon tubing. For dissolved metal contents, water samples were filtered in situ using a 0.45 μm filter capsule connected in-line with Tygon tubes. Filtered and non-filtered water samples were collected into low-density polyethylene (LDPE) bottles at each sampling site. After water collection, temperature, pH, salinity, and dissolved oxygen (DO) were measured in situ by using an electrochemical hand-held device(Hach Co., Loveland, CO, USA). In the laboratory, to determine total and dissolved metal, samples (500 mL) were acidified with HNO_3_ (2 mL/L), kept at room temperature for a week and, subsequently, stored at −20 °C until analysis. Non-filtered water was also collected for density and suspended solids (SS) determinations, and filtered water (acidified with HCl at 2 mL/L) was used for dissolved organic carbon (DOC) analysis by means of a TOC analyser (Shimadzu, Columbia, MD, USA). Organic matter content in SS was determined via weight loss using a muffle furnace(Nabertherm, Lilienthal, Germany) at 550 °C.

The content of total and dissolved metals in water was measured by differential pulse anodic stripping voltammetry (DPASV) after acid digestion. Samples of 45 mL were digested with 0.2 mL of 65% HNO_3_ and 0.125 mL HClO_4_ in a closed Teflon reactor (BRAND, 1305 38, Wertheim, Germany) for 8 h at 120 °C, cooled, and diluted with Milli-Q (Millipore, Burlington, MA, USA) deionised water up to 50 mL in a volumetric flask. Due to the low metal concentrations obtained for the dissolved content analyses, determinations of dissolved labile and moderately labile fractions could not be performed. Thus, the speciation studies had to be limited to the fractionation assessment of dissolved and particulate metal contents. This latter fraction was calculated by the difference between total and dissolved fractions.

The limits of detection of the metal analysis in the water samples are presented in Table S3 of the Supplementary Material.

#### 2.3.2. Sediment

Surface sediment samples were collected using a Van Veen grab sampler (KC Denmark, Silkeborg, Denmark) and immediately stored in polyethylene bags. In the laboratory, sediment samples were preserved at −20 °C until pretreatment. They were thawed, oven-dried at 40 °C for 24 h, ground in an agate mortar, sieved through a stainless steel mesh to obtain fine particle-size fractions (<0.063 mm), and stored in polyethylene bottles at room temperature until analysis.

Metal fractions in sediments were obtained by using the three-step sequential extraction procedure proposed by the European Community Bureau of Reference (BCR, now replaced by the Standards, Measurement, and Testing Programme). This widely recognised method of fractionation was developed and improved in order to standardise and harmonise the various schemes described in the literature [31,32,33,34,35,36,37] and provides chemical information about the extractable acids (water-soluble, exchangeable, and bound to metal carbonates), reducible (bound to Fe-Mn oxides), oxidisable (bound to sulphides and organic matter), and residual (inert) metal fractions in the sediment [37,38,39,40].

Total metal content in the sediment samples as well as the analysis based on the modified three-step BCR extraction procedure, were performed as described in Table S4 of the Supplementary Material. After applying this fractionation scheme, four different fractions can be obtained in the sediments: exchangeable (F1), reducible (F2), oxidisable (F3), and residual (F4) fractions. All metal extracts obtained from the procedures were stored at 4 °C into acid-washed polyethylene bottles until analysis by inductively coupled plasma-mass spectrometry (ICP-MS).

The organic matter content in the sediments (OM in S) was calculated by the weight loss of dried samples by combustion at 550 °C.

The limits of detection of the metal analysis for the total content and the different fractions of BCR procedure in sediments are shown in Table S3 of the Supplementary Material.

### 2.4. Quality Control and Quality Assurance

All experimental procedures were carried out using latex gloves and a second pair of disposable PE gloves, which are free of trace metals and usually used in clean rooms. LDPE bottles for water collection were pre-cleaned with 3 mol/L HCl and immersed in a drum container filled with 0.1 mol/L HCl for 6 weeks, then rinsed six times with ultrapure water before they were air dried in a laminar flow hood. Plastic and glass labware were cleaned using a 2 mol/L nitric acid bath overnight, followed by rinsing with ultrapure water and air drying in a laminar flow hood. The materials were finally sealed in polyethylene bags until use. Each sample was prepared and processed in duplicate and analysed in three replicates (n = 3) to ensure the reliability of the methods and measurements. In all cases, blank samples were performed following the same protocols described for samples. Standard solutions for metal calibration curves were prepared in matrices similar to the samples. Standards and blanks were also run between every 10 sets of samples for quality control of the measurements. The average values of the relative standard deviations (%RSD)—obtained from the three replicates of standards and samples—were most often < 10%. The limits of detection (LD) of the metal analysis were determined (defined as *3·s/m*, where *s* is the standard deviation of 10 blank measurements and *m* is the slope of the calibration curve [41]) (Table S3 of the Supplementary Material). The following certified reference materials were analysed following the same procedures as for the samples obtaining successful recoveries rates (Table S5 of the Supplementary Material): estuarine water reference material BCR-505 acidified to pH 1.6 (recoveries of 88.8–106.9%), from the European Community Bureau of Reference (BCR); estuarine sediment NIST-SRM 1646a for total metal content (recoveries of 83.3–101.4%), and lake sediment BCR-701 for BCR procedure (recoveries ranges of 89.5–116.8, 73.8–95.9 and 94.9–109.5% for F1, F2, and F3 fractions), purchased from National Institute of Standards and Technology (NIST) and the BCR, respectively.

### 2.5. Statistical Software

Statistical analyses of the obtained data were performed using STATISTICA 7 software package (STATSOFT 2004, Inc., Tulsa, OK, USA). First, Levene and Brown–Forsythe tests were used to measure the homogeneity of the data, and the normality of results was checked by the Shapiro–Wilk test (n < 30) or the Kolmogorov–Smirnov test (n > 30). Some data were neither homogeneous nor normally distributed even when they were mathematically transformed (log x, log (1 + x), 1/x, 1/(1 + x), x^2^). In these cases, a series of non-parametric tests were carried out. The evaluation of significant differences of analysed metals levels within samplings and sites for the different samples were estimated using the parametric one-way ANOVA or the non-parametric Kruskal–Wallis test and the multiple comparison tests. The Pearson matrix was used to determine the correlation between the concentrations of the pollutants in the different environmental compartments for homogeneous and normal data, while the Spearman’s Rank correlation was employed for non-homogeneous and non-normal distributions. Results of testing were considered significant at *p* ≤ 0.05.

## 3. Results and Discussion

### 3.1. Physico-Chemical Parameters of Abiotic Samples

During the different samplings, the physico-chemical parameters of samples were determined. Some parameters of water samples were measured in situ during the sampling stage (temperature, pH, salinity and DO) while others were determined in the laboratory (DOC in water, SS concentration, organic matter in SS, and organic matter in sediments) (Table 1). Water parameters were compared to the quality objectives published in Appendix II of the Official Bulletin of the Andalusian Autonomous Government nº 27, 1997 [42], provided as a guide to quality-assurance of Andalusian coastal waters.

Temperature values ranged between 14.3–22.1 °C, with the lowest values in Sampling 3 (2nd autumn). The recorded pH values were in the range of 7.0–8.6, so these waters can be classified as slightly alkaline. Salinity values ranged between 29.3 and 37.2‰, showing the lowest values in sampling 4 (2nd spring). However, these values did not exceed those established for coastal waters [42]. The DO showed lower values in samplings 1 and 3 (autumn season), when the seasonal biological activity is over and the degradation processes could be higher. This seasonal trend could be explained by the fact that the photosynthetic processes in this area show their maximum activity from April to July. Some of the DO values were lower than the mandatory minimum value proposed by Andalusian Government (70% sat) [42]. The SS concentrations ranged from 0.014 to 0.040 g/L, showing the lowest values in sampling 2 (1st spring) and the highest for sampling 1 (1st autumn), in which site 5 exceeded the mandatory value proposed by Andalusian Government (0.032 g/L) [42]. A seasonal trend was also observed for organic matter content in SS with higher values in samplings 1 and 3 (autumn season), which is in agreement with the low DO levels, as organic matter breakdown involves oxygen consumption. The DOC content in water was quite remarkable, also in sampling 1 at site 5 (6.23 mg/L) exceeding the threshold value of 3 mg/L [42]. Regarding the organic matter content in sediments, higher average values corresponded to samplings 1 and 3 (autumn season), which could be influenced by the natural occurrence of biological activity over the summer or the increase in tourist population during this season. The values were within the normal ranges found in sediments of this type of ecosystems [43,44].

Therefore, DO in water, organic matter in SS, and organic matter in sediments presented a seasonal trend. Temperature and DO values were compared to others obtained in the same bay, where temperature values were found to be similar and DO values were slightly higher [45].

Spearman correlation analysis applied to these parameters showed, as significant results, that salinity and DO were negatively correlated (*p* < 0.05; R_Spearman_ = −0.74153), as the presence of dissolved solids (providing salinity) usually reduces the solubility of gases in water. DO showed a negative correlation with organic matter in sediments (*p* < 0.05; R_Spearman_ = −0.74567), but DOC showed a high correlation with SS (*p* < 0.05; R_Spearman_ = 0.72658).

### 3.2. Metal Content in Water Samples

**Figure 2:**
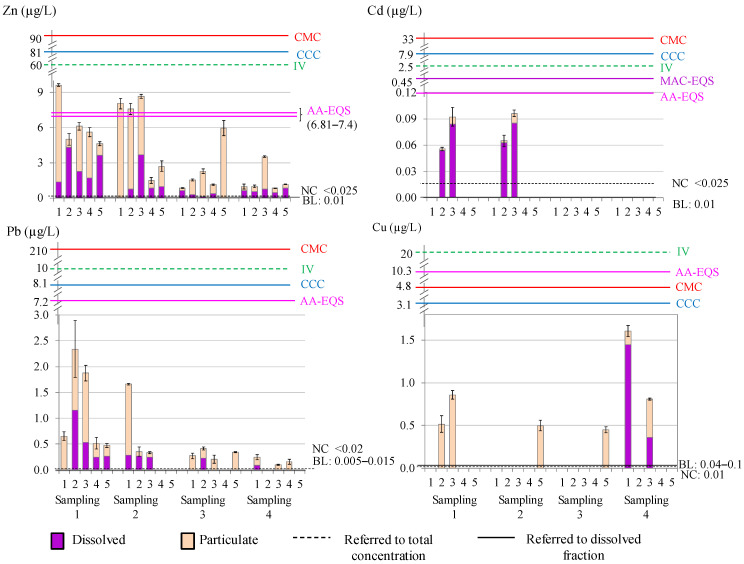
Metal content and reference values in water (BL: background level; NC: natural concentration; IV: imperative value, for total content; CCC: criteria continuous concentration; CMC: criteria maximum concentration, for dissolved content; AA-EQS: annual average value; MAC-EQS: maximum allowable concentration) (*Note*: sites with absence of data were below the LD).

Total metal content and the fractionation into dissolved and particulate phases are summarised in Figure 2. The total metal concentrations (expressed as mean ± S.D.) were: 3.93 ± 2.96 μg/L for Zn, 0.015 ± 0.033 μg/L for Cd, 0.50 ± 0.67 μg/L for Pb, and 0.24 ± 0.43 μg/L for Cu (Zn > Pb > Cu > Cd). The dissolved contents were: 1.21 ± 1.28 μg/L for Zn, 0.014 ± 0.030 μg/L for Cd, 0.17 ± 0.28 μg/L for Pb, and 0.09 ± 0.33 μg/L for Cu (Zn > Pb > Cu > Cd). The particulate contents were: 2.73 ± 2.73 μg/L for Zn, <LD μg/L for Cd, 0.33 ± 0.45 μg/L for Pb, and 0.15 ± 0.26 μg/L for Cu (Zn > Pb > Cu > Cd).

Average concentrations of Zn were higher in samplings 1 and 2. Site 1 (control site) presented higher concentrations than expected in the two first samplings, which could be related to shipwrecks and a few fuel spills in this area since it is a maritime transport area. Site 3 also presented high concentrations compared to the other sites, probably due to the presence of the steel manufacturing and thermal plants, and Palmones Estuary; while the concentrations at site 5 can be attributed to port activity in the area. Cd was detected at low concentrations, mostly in its dissolved form, in samplings 1 and 2 at sites 2 and 3, probably due to port and industrial activities. Regarding Pb total content, the highest concentrations were also detected in samplings 1 and 2, showing maximum values at sites 1, 2, and 3 in sampling 1, which could be associated with spills and air pollution contributions to the bay from dense industrial emissions in the area. These concentrations seem to be spread over time as seen in the low concentrations detected in the two subsequent samplings. Cu content was fairly heterogeneous, with higher values in samplings 1 (sites 2, 3) sampling 4 (sites 1, 3), and site 5 for samplings 2 and 3. By conducting, non-parametric Kruskal–Wallis ANOVA analyses, no seasonal trend was observed for any metal in the dissolved phase; but seasonal differences were observed for total and particulate Pb between spring and autumn (*p* values of 0.0272 and 0.0120, respectively).

The distribution coefficient of a metal between the particulate and dissolved phases, is defined as the ratio of particulate metal concentration to dissolved metal concentration: K_d_ (L/kg) = [M_particulate_] (mg/kg)/[M_dissolved_] (mg/L) [46]. The value of log K_d_ is usually used to evaluate the balance of heavy metals partitioning between both phases. A high value of log K_d_ indicates a higher affinity of the metal for suspended particles, while a low value means that the metal is mainly in its dissolved form [47]. The different partitioning behaviours depend on the interactions of the chemical constituents between the suspended particles and water, which result from a variety of physical, chemical, and biological processes [48]. High particle reactivity allows the association of the metal with particulate matter, and low particle reactivity, together with a strong potential to form stable complexes, can cause the metal to remain in the dissolved phase [49]. The log K_d_ (L/kg) ranges for the Algeciras Bay were: 3.80–5.89, 3.19–3.91, 4.26–5.53, and 3.73–4.73 for Zn, Cd, Pb, and Cu, respectively. Lower values for Cd supported the fact that this metal remains in its dissolved form (>88%), making this metal more bioavailable to the aquatic biota, although it occurs at low concentrations. Zn and Pb presented higher log K_d_ values, and Cu to a lesser extent, revealing greater affinity for the particulate phase and, predictable, lower toxic potential. In addition, the total contents of Zn, Pb, and Cu in water were highly correlated with the particulate phases (R_Spearman_ = 0.9098, 0.8702, and 0.9520, respectively), which indicates a certain tendency of these metals to precipitate in this ecosystem. The log K_d_ for Zn and Pb showed slight negative correlations with suspended matter, which can be explained by the “particle concentration effect” that had been attributed to heterogeneity effects of particle size and composition [50]. Therefore, it must be concluded that the percentage of dissolved metal versus particulate could be arranged in the following order: Cd >> Zn > Cu > Pb (average values: 93.3%, 39.4%, 29.2%, and 22.5%, respectively). The partitioning coefficients were compared with those found in other bays and estuaries (Table S6 of Supplementary Material) [46,47,51,52,53,54,55,56,57,58,59]. The ranges of log K_d_ for Zn were similar except those higher, which were found in Cadiz Bay (Spain) and the Dakar coast and Saint Louis Estuary (Africa) [54,55]. For Cd, results were also similar except in Sagami Bay (Japan), Zhanjiang Bay (China), and Masan Bay (Korea) with higher values [46,47,58]. Similar values were also found for Pb with the exception of the higher values found in the Dakar coast and Saint Louis Estuary (Africa) [55]. For Cu, the values were similar in these bays and estuaries. These findings indicate that most comparative studies show similar metal availability in water.

#### Comparison with Guide Levels and Other Ecosystems

In order to assess the possible impacts on the environment and health, the levels of heavy metals in the waters were compared with reported reference values for coastal and seawaters (Figure 2): (i) background level (BL) referred to the dissolved content [60]; (ii) natural concentration (NC) referred to the total concentration [61]; (iii) imperative values of the Andalusian Government (IV) for the total metal content for limited and non-limited areas (considering the Algeciras Bay as a non-limited area) [42]; (iv) quality guidelines for the protection of aquatic life proposed by the Environmental Protection Agency (EPA) in saltwater [62]—Criteria Maximum Concentration (CMC) for acute contamination and Criteria Continuous Concentration (CCC) for chronic contamination—both referring to the dissolved content; and (v) environmental quality standards presented in the Water Frame Directive (Directive 2000/60/EC of the European Parliament) [63] (AA-EQS for annual average and MAC-EQS for maximum allowable concentration, referred to the dissolved content). Environmental standards for Zn and Cu were calculated as described in the directive. All detected average metal concentrations exceeded the BL and NC values. Higher concentrations were found for Zn in samplings 1 and 2 (sites 1, 2, and 3). For Cd, the concentrations were close to the AA-EQS value. Therefore, it can be stated that the levels of metals found in waters did not compromise the safety of aquatic life in the ecosystem, since concentrations only exceeded the BL and NC values, and most of the limit values were not reached.

The total metal concentrations found in the waters of the Algeciras Bay were compared with those of other similar studies (Table 2). Positive values in red mean the number of times our results are higher compared to the others, and negative green values mean the number of times they are lower.

Not very different values were obtained in the same bay by Morillo et al. [28]. In general terms, the Algeciras Bay show concentrations similar to or lower than those of the other sites in Spain, becoming up to approximately 79, 187, and 317 times lower than those reported for Zn, Cd, and Cu, respectively, in the highly polluted area of the Huelva Estuary [28]. However, Zn values were slightly higher in Algeciras than in the Guadalquivir and Guadiana estuaries (1.5 and 4.5 times) [64] or the Bilbao harbour (1.4 times) [65]. Similarly, Pb levels were higher than those reported for the Guadalquivir, Guadiana, and Tinto-Odiel estuaries (13.0, 14.6, and 1.3 times, respectively) [64] and the Vigo, Bilbao, and Pasajes harbours (2.7, 3.4, and 9.1 times, respectively) [65]. Compared with other international ecosystems, values from Algeciras Bay were lower with very marked differences in some cases.

### 3.3. Metal Content in Sediment Samples

**Figure 3:**
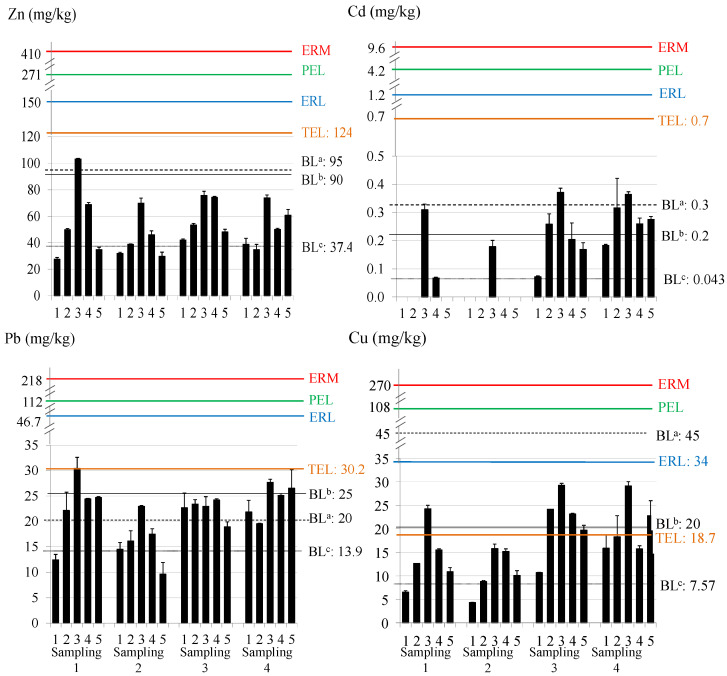
Total metal content and guideline values in sediment. BL^a,b,c^: background levels proposed by Turekian and Wedepohl [78], OSPAR [79], and Besada et al. [80]; ERM: effects range medium; ERL: effects range low; PEL: probable effect level; TEL: threshold effect level. Note: sites with absence of data were below the LD.

The total metal content in sediments is presented in Figure 3. The average metal concentrations (expressed as mean ± S.D.) were: 52.61 ± 19.28 mg/kg for Zn; 0.15 ± 0.13 mg/kg for Cd; 21.38 ± 5.10 mg/kg for Pb; and 16.63 ± 6.96 mg/kg for Cu (Zn > Pb ≈ Cu > Cd).

This trend is similar to that obtained in water samples. In general, sites 3 and 4 presented higher total metal content in the sediments, due to their proximity to steel and thermal production plants, where heavy metals such as Zn, Pb, and Cu are widely used. Regarding the temporal distribution, sampling 2 presented the lowest average concentrations, while samplings 3 and 4 showed, in general, higher average concentrations. In the case of Cd, some samples presented content below the LD at some sites in samplings 1 and 2.

**Figure 4:**
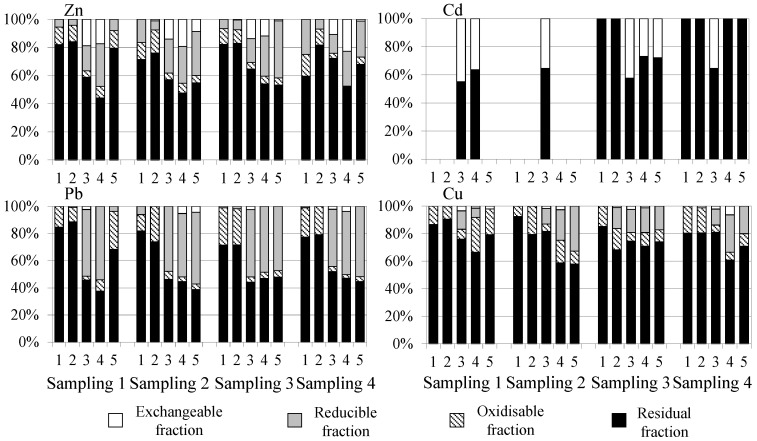
Distribution of heavy metals in sediment from Algeciras Bay obtained by BCR sequential chemical extraction procedure. Note: sites with absence of data were below the LD.

The distribution of metals in the sediment (in %) obtained by the BCR sequential extraction procedure is shown in Figure 4.

The content of heavy metals chemical fractions depends on factors such as pH, oxidation-reduction potential, particle size, iron and manganese oxides, organic carbon, and acid volatile sulphides [81]. Most of the metals (especially Cu with a range of 58 to 92%) were mainly found in the residual fraction, where the elements are strongly bound to silicates of the crystalline structure of the sediment and are relatively stable, with low mobility and less bioavailability [82]. The contents of the oxidisable fractions of Pb and Cu (3–27% and 5–25%, respectively) may be due to the affinity of these metals for the organic matter that leads to the formation of complexes between them. Cu has tendency to form stable organo-Cu complexes due to its affinity towards humic substances, among other organic ligands [83]. The release of these elements is caused by the degradation of organic matter or oxidation of sulphides to sulphates under aerobic conditions [23]. At some points, Zn, Pb, and Cu presented higher percentages in the reducible fraction (4–40%, 0–54% and 0–33%, respectively) than Cd (no presented reducible fraction content). This fact may be associated with the ability of oxides of Mn and Fe to bind and form complexes with Zn, Pb, and Cu, which can be mobilised to the water column under anoxic (reducing) conditions and be captured by benthic organisms [84]. Finally, Cd showed a high percentage of exchangeable fraction (0–45%) in some sites (3, 4, and 5). In soils and sediments, Cd is often bound to the labile or moderately labile exchangeable, carbonate and hydrous oxide fractions than other heavy metals such as Pb and Cu, which are more strongly bound to the organic and sulphidic fractions [85]. The strong association of Cd with the carbonate fraction is probably due to the similar ionic radius of Cd (0.97 Å) and Ca (0.99 Å), enabling the replacement of Ca by Cd in the calcite crystal [86]. Zn also showed some mobility in the sediments (0–23%) at sites 3 and 4, so it can be expected that the exchange of this element between the sediment and the water column takes place easily [87]. The metals associated with this fraction can present high mobility due to their low binding capacity to sediments. They can be easily released into the water column due to changes in pH and ionic strength, considering this fraction as bioavailable [88,89]. Therefore, metal concentrations in the residual fraction are considered a non-mobile fraction, while the non-residual fractions are considered mobile, determining the potential risk of these elements to the surrounding environment and aquatic life [90]. The highest availabilities were for Cd, due to its high percentage of exchangeable fraction (F1), and for Pb due to both the exchangeable (F1) and reducible fractions (F2). Even though Cd was the most available metal due to F1, which may pose high health risks compared to the other fractions, it can be assumed that Cd will not cause serious danger because of its low levels. However, Pb could have a higher impact than Cd.

Spearman correlations in sediments were significant (at *p* < 0.05) for total Zn and Pb (R = 0.74736), Zn and Cu (R = 0.81353), and Cd and Cu (R = 0.88807). There were no notable differences between the metal content in the sediments and the physico-chemical parameters, nor between the metal content in sediments and waters. In relation to the BCR fractionation studies, the following positive correlations (R > 0.75) were found: exchangeable Zn-Cd (*p* = 0.80586), reducible Zn-Pb (*p* = 0.82530), oxidisable Zn-Pb (*p* = 0.91729), oxidisable Zn-Cu (*p* = 0.82406), oxidisable Pb-Cu (*p* = 0.75789), residual Zn-Pb (*p* = 0.79548), residual Zn-Cu (*p* = 0.84060), exchangeable Zn-reducible Pb (*p* = 0.83093), and oxidisable Pb-residual Pb (*p* = 0.88421). This reveals the similar geochemical behaviour of some elements. The content of total Zn in sediments highly correlated with the exchangeable fraction (R_Spearman_ = 0.7621), total Cu with the reducible phase (R_Spearman_ = 0.7496), and oxidisable Pb with the residual fraction (R_Spearman_ = 0.7955). This information reveals that Zn and Cu are the metals most associated with the most available fractions and can be resuspended from the sediment to the ecosystem under certain environmental conditions.

Non-parametric Kruskal–Wallis ANOVA analyses (the data set could not be normalised) were applied to the metal content data (total and fractions) in order to understand significant differences between sampling sites. Several significant differences were found: (a) for sites: total Zn and Cu (sites 1 and 3, *p* = 0.01538 and *p* = 0.04123, respectively), exchangeable Cd (sites 1 and 3, *p* = 0.05977 and sites 2 and 3, *p* = 0.05977), exchangeable Zn (sites 1 and 3, *p* = 0.01250), residual Zn (sites 2 and 4, *p* = 0.01250); reducible Pb (sites 2 and 3, *p* = 0.04972 and sites 2 and 4, *p* = 0.04972), residual Pb (sites 2 and 4, *p* = 0.03408), exchangeable Cu (sites 1 and 3, *p* = 0.02305); (b) for samplings: total Cd (samplings 2 and 4, *p* = 0.02767), residual Cd (samplings 2 and 3, *p* = 0.02767). Seasonal differences were only found for oxidisable Cu (*p* = 0.04125). The most significant differences were observed between sites 1 and 3 for Zn, corresponding to the control and one of the industrialised sites.

#### 3.3.1. Comparison with Guide Levels and Other Ecosystems

The total content in the sediments of the Algeciras Bay was compared with some guideline values in order to assess the environmental status of this coastal area (see Figure 3). The background levels (BL) proposed by Turekian and Wedepohl [78], OSPAR [79], and Besada et al. (local background levels for metals in the Gulf of Cádiz) [80] were used. Besada et al. proposed background metal concentrations for the Gulf of Cadiz using surface sediments due to the need to have guide levels for sediments based on their own composition and location. On the other hand, the potential ecological risk of sediments can be evaluated using the ranges developed by the US National Oceanic and Atmospheric Administration (NOAA) based on the ERL (Effects Range Low) and ERM (Effects Range Medium) values. Metal concentrations below the ERL mean that adverse biological effects are rarely observed or predicted, while above the ERM, adverse biological effects are usually or always observed [91]. In the same way, environmental criteria based on TEL (Threshold Effect Level) and PEL (Probable Effect Level) values were also used. Concentrations below the TEL mean that adverse effects rarely occur and are often expected if levels are above the PEL [92]. For Zn, most of the results exceeded the background level proposed by Besada et al. (37.4 mg/kg) and site 3 also exceeded the highest BL (95 mg/kg). For Cd, all values exceeded Besada BL (0.043 mg/kg) and, in some cases, also the others; in samplings 3 and 4, the concentration at site 3 exceeded the BL of Turekian and Wedepohl (0.3 mg/mg). For Pb, most sites exceeded the BL of Besada et al. and Turekian and Wedepohl (13.9 and 20 mg/kg), and some sites reached the BL of OSPAR (25 mg/kg). Site 3 in sampling 1 reached the TEL value for Pb (30.2 mg/kg) and some sites in sampling 4 were close to it. For Cu, most concentrations exceeded Besada et al.’s BL (7.57 mg/kg); site 3 in sampling 1 and most sites in samplings 3 and 4 exceeded TEL (18.7 mg/kg) and the OSPAR’s BL (20 mg/kg). Although episodes of marine toxicity due to these concentrations are not expected, environmental adverse effects can occur regarding Cu and Pb levels, principally in site 3.

A comparison of the average total metal concentrations in sediments from different sites is shown in Table 3. Positive values in red mean the number of times our results are higher compared to the others, and negative green values mean the number of times they are lower. In general, higher Pb values have been found in the sediments of the Algeciras Bay than in other Spanish ecosystems [54,93,94,95,96,97,98], up to 6.4 times higher than Galician coast) [94], and approximately 3.5 times higher than Bay of Biscay and Valencian coastline [97,98]. The Huelva estuary showed values much higher values than Algeciras Bay (23.4, 66.7, 26.7, and 125 times higher for Zn, Cd, Pb, and Cu, respectively) [54]. For Zn, values were also higher in comparison to Bay of Biscay (2.5 times higher) [97]; and for Cu, 7.5, 4.0, and 4.7 times higher than in Galician coast, Bay of Biscay, and Valencian coastline, respectively [94,97,98]. However, when compared with bays located in the Mediterranean Sea, the results from Algeciras Bay are lower than those for Thessaloniki Bay (Greece) [99], Golfe-Juan Bay (France) [100], and Edremit Bay (Turkey) [101] for all metals. Finally, when comparing with other continents [73,74,102,103], Algeciras Bay values for Pb were notably higher than those from Red Sea Coast (Saudi Arabia) [73] (5.5 times) and Qua Iboe Estuary (Nigeria) [74] (4.1 times), and somewhat higher than Todos Santos Bay (Mexico) (1.8 times) [102].

#### 3.3.2. Ecological Risk Assessment of Sediment Quality Using Pollution Indexes

The assessment of sediment pollution was estimated by using different pollution indexes such as the enrichment factor (EF), contamination factor (CF), and geoaccumulation index (I_geo_) [104]. The possible presence of anthropogenic inputs was evaluated using as background levels, the local levels reported for the Gulf of Cadiz (Zn: 37.4, Cd: 0.043, Pb: 13.9, Cu: 7.57, Fe: 8800 mg/kg dry weight) [80] instead of the average metal concentrations in continental shales (sedimentary rocks) [78] and average crustal abundance [105] commonly used to provide elemental background concentrations in these calculations.

**Figure 5:**
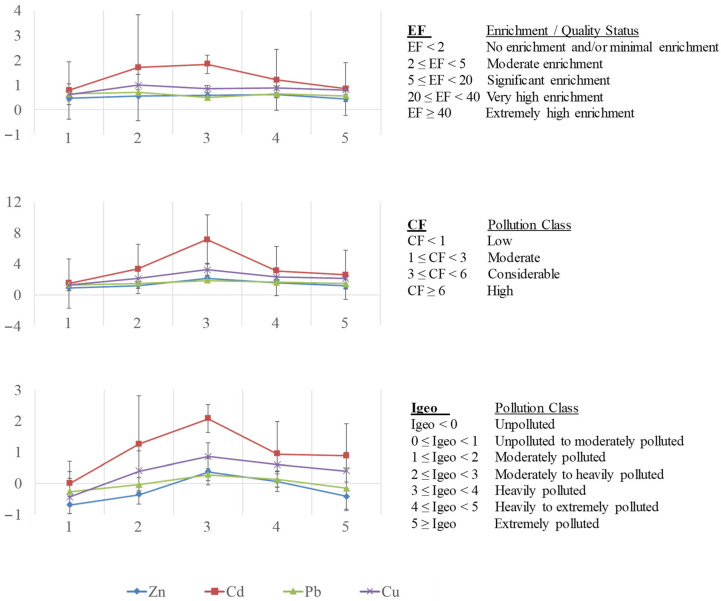
Mean values for enrichment factor (EF), contamination factor (CF) and geoaccumulation index (I_geo_) in sediments at five sampling sites of Algeciras Bay.

The indexes were calculated using the following equations and the classification of sediment quality according to them is given in Figure 5:

(1) $$EF=\frac{{\left(M/Fe\right)}_{sample}}{{\left(M/Fe\right)}_{background}}\;$$

(2) $$CF=\frac{M_{sample}}{M_{background}}$$

(3) $$I_{geo}={log}_{2}\left(\frac{M_{sample}}{{1.5\cdot M}_{background}}\right)$$

In Equation (1) [106], (*M/Fe*)_s*ample*_ is the ratio between the concentrations of metal (M) and Fe in the sample, and (*M/Fe*)*_background_* is the ratio between background concentrations of metal and Fe. This index was calculated by normalising the concentration of a metal in the sediment to the concentration of a reference element. Fe was used as a reference element since its concentration in the sample is influenced exclusively by crystalline sources, which means that it is not anthropogenically affected and is characterised by low variability of occurrence and fine particles [107,108,109,110].

In Equation (2) [111], *M_sample_* is the concentration of the metal in the sediment and *M_background_* is the reference concentration found in sedimentary rocks.

In Equation (3) [112], *M_sample_* is the measured concentration of the metal and *M_background_* is the background concentration. The factor 1.5 includes possible variations in background values due to lithological variations.

Figure 5 shows these pollution indexes for each metal. All EF values [113] were <2, indicating “no enrichment or minimal enrichment” of these sediments. Nevertheless, values higher than 1 were found for Cd at site 3 (1.86), site 2 (1.55), and site 4 (1.16), suggesting greater enrichment of the sediments by this metal at these sites influenced by urban and industrial activities. Regarding CF values [114], the highest values were again obtained for Cd at site 3 (7.12, “high pollution”, CF ≥ 6), site 2 (3.34), and site 4 (3.09), as well as for Cu at site 3 (3.25), classified as “considerable pollution” (3 ≤ CF < 6). The rest of the values presented “moderate pollution” (1 ≤ CF < 3), being the lower value Zn at site 1 (0.94). I_geo_ values [112,114] were also higher for Cd at site 3 (2.25, “moderately to heavily polluted”, 2 ≤ I_geo_ < 3), site 2 (1.15), and site 4 (1.04), and Cu at site 3 (1.11), considered as “moderately polluted” (1 ≤ I_geo_ < 2). The rest of the values ranged between unpolluted and moderately polluted (I_geo_ < 1).

Hence, taking into account the values of CF for sediment, site 3 was considered as highly polluted for Cd; while sites 2 and 4 for Cd, as well as site 3 for Cu, were classified as moderately polluted. The highest I_geo_ values were obtained at site 3 (moderately to heavily polluted), and secondly, at sites 2 and 4 for Cd, and site 3 for Cu (considered as moderately polluted). This ecological risk assessment revealed that the sediments presented as polluted with respect to all metals: high for Cd in site 3; considerable for Cd at sites 2 and 4, as well as for Cu at 3; and low or moderate pollution by Pb and Zn, indicating the influence of anthropogenic activities, especially at site 3, characterised with the greatest urban and industrial impact in the area.

## 4. Conclusions

The trace metal levels in both water and sediment samples from Algeciras Bay exhibited a similar trend: Zn > Pb ≈ Cu > Cd. While there were minimal seasonal variations in water and sediments, spatial variations in sediment metal content were observed among sites, particularly with sites 3 and 4 showing higher metal contents due to proximity to steel and thermal production plants. Total Zn concentrations were significantly different between sites 1 and 3. Zn had the highest total content in water, especially at sites 1, 2, and 3, while Cd content was low but mostly found in the dissolved phase, making it more bioavailable. Other metals showed a high correlation between total and particulate content, promoting precipitation and sorption processes. Comparison with guideline values indicated that metal concentrations in water did not significantly compromise aquatic life safety, except for Zn and Cd, which approached the AA-EQS level (0.12 µg/L for the dissolved fraction). Strong currents and deep waters in the area could disperse and reduce metal contents. Compared with other ecosystems, the Algeciras Bay showed lower levels for all studied metals, except for Pb in some samples from sites of importance due to their anthropogenic impact (average: 0.50 ± 0.67 µg/L).

In sediments, sites 3 and 4, located near local industries, exceeded the threshold effect level (TEL) for Cu and approached it for Pb, at some samplings. BCR extraction revealed high availabilities for Cd and Pb due to their exchangeable and reducible fractions. These results are crucial as these fractions could resuspend in water, posing potential risks to marine fauna and human health. Correlations in sediments were found between total Zn and Cu and their exchangeable and reducible fractions, suggesting proportional increases in the more labile fractions. Compared to other ecosystems, Algeciras Bay showed lower metal levels, except for Pb (average: 21.4 ± 5.1 mg/kg) and Cu (average: 16.6 ± 7.0) in some samples with anthropogenic impact. Ecological risk assessment indicated that sampling site 3 was the most polluted by metals, particularly for Cd (CF = 7.12 and I_geo_ = 2.25). These interesting results from the abiotic samples were completed with the study of different fish species (benthic and benthopelagic) from the same sites in *Ecological status of Algeciras Bay, in a highly anthropised area in south-western Europe, through metal assessment*—*Part II: Biotic samples*, as an integrative way to assess the ecological status of this significant bay. This approach to the ecological status of an aquatic ecosystem can serve as a guide and/or comparison for application in other areas under high anthropogenic pressure that require such studies.

## Figures and Tables

**Table 1:** Physico-chemical parameters of abiotic samples.

Sampling	Site ^a^	Water					SS ^d^		Sediment
		T (°C)	pH	Salinity (‰)	DO ^b^ (% sat)	DOC ^c^ (mg/L)	SS (g/L)	OM ^e^ (%)	OM (%)
1 (1st autumn)	1	21.2	8.34	35.6	47.7	1.72	0.027	32.9	16.1
	2	22.1	8.27	35.5	48.3	1.52	0.024	17.2	17.8
	3	20.1	8.25	37.2	43.7	1.39	0.024	26.5	12.3
	4	21.3	8.24	35.2	45.2	1.53	0.032	25.7	11.6
	5	21.0	7.03	34.4	44.5	6.23	0.040	35.0	10.2
2 (1st spring)	1	18.3	8.42	34.8	85.8	0.41	0.014	12.8	2.6
	2	19.7	8.42	33.9	90.4	0.59	0.015	18.2	5.3
	3	20.2	8.56	32.4	88.8	0.67	0.016	13.4	1.8
	4	20.9	8.51	33.8	89.6	0.72	0.015	20.2	1.7
	5	18.9	8.58	33.5	88.5	0.56	0.016	19.0	1.5
3 (2nd autumn)	1	14.9	7.98	32.9	77.5	1.74	0.029	19.4	4.1
	2	15.9	8.03	32.8	75.1	1.03	0.017	12.6	7.6
	3	15.4	8.06	32.2	67.7	0.66	0.022	17.9	6.8
	4	15.3	8.04	31.2	73.5	1.67	0.024	21.4	7.6
	5	14.3	8.07	32.4	66.8	1.52	0.019	17.8	15.7
4 (2nd spring)	1	18.2	7.11	30.1	88.0	0.92	0.021	12.8	3.1
	2	18.3	6.96	29.3	83.5	2.51	0.022	16.1	5.7
	3	18.2	7.58	30.3	95.5	0.41	0.023	14.6	2.4
	4	18.6	7.37	29.5	88.4	1.05	0.022	12.6	4.5
	5	18.5	7.42	29.7	94.0	1.14	0.020	7.1	4.4

^a^ Sampling site: 1: Getares, 2: Isla Verde, 3: Palmones, 4: Guadarranque, 5: Puente Mayorga; ^b^ DO (% saturation): dissolved oxygen; ^c^ DOC: dissolved organic carbon; ^d^ SS: suspended solids; ^e^ OM: organic matter.

**Table 2:** Average values of total metal concentrations (µg/L) in waters from different aquatic ecosystems and their comparison with those from this study of the Algeciras Bay ^a^.

Site	Zn	Cd	Pb	Cu	Reference
Algeciras Bay	3.93	0.015	0.5	0.24	This study
Algeciras Bay	9.8 (−2.5)	0.02 (−1.3)	-	0.5 (−2.1)	[28]
Huelva Estuary	309 (−78.6)	2.8 (−186.7)	-	76 (−316.7)	
Huelva Estuary	167.17 (−42.5)	2.8 (−186.7)	6.27 (−12.5)	46.38 (−193.3)	[54]
Cádiz Bay	20.98 (−5.3)	0.18 (−12.0)	5.97 (−11.9)	6.29 (−26.2)	
Guadalquivir Estuary	2.62 (+1.5)	0.047 (−3.1)	0.038 (+13.0)	2.32 (−9.7)	[64]
Guadiana Estuary	0.87 (+4.5)	0.026 (−1.7)	0.034 (+14.6)	0.71 (−2.9)	
Tinto-Odiel Estuary	19.29 (−4.9)	0.29 (−19.2)	0.38 (+1.3)	5.96 (−24.8)	
Vigo Harbour	5.42 (−1.4)	0.017 (−1.1)	0.18 (+2.7)	1.73 (−7.2)	[65]
Bilbao Harbour	2.72 (+1.4)	0.021 (−1.4)	0.15 (+3.4)	1.06 (−4.4)	
Pasajes Harbour	8.24 (−2.1)	0.027 (−1.8)	0.055 (+9.1)	0.32 (−1.3)	
Nizampatnam Bay (India)	25.54 (−6.5)	12.14 (−809)	9.72 (−19.4)	20.68 (−86.2)	[66]
Jinzhou Bay	12.34 (−3.1)	0.88 (−58.5)	0.08 (+6.6)	2.45 (−10.2)	[67]
Zhanjiang Bay	12.64 (−3.2)	0.12 (−7.8)	0.23 (+2.2)	4.4 (−18.3)	[47]
Meishan Bay	101.53 (−25.8)	4.68 (−311.8)	1.50 (−3.0)	5.38 (−22.4)	[68]
Beibu Gulf	10 (−2.5)	0.17 (−11.3)	0.71 (−1.4)	3.03 (−12.6)	[69]
Daya Bay	4.08 (1.0)	0.086 (−5.7)	0.603 (−1.2)	1.61 (−6.7)	[70]
Xiangshan Bay	16.8 (−4.3)	0.22 (−14.7)	1.93 (−3.9)	3.4 (−14.2)	[71]
Liaodong Bay	17.76 (−4.5)	0.66 (−44)	3.98 (−8)	2.86 (−11.9)	[72]
Red Sea Coast, Jizan (Saudi Arabia)	3.58 (+1.1)	0.17 (−11.3)	0.56 (−1.1)	7.85 (−32.7)	[73]
Qua Iboe Estuary (Nigeria)	2.2 (+1.8)	1.8 (−120)	2.4 (−4.8)	-	[74]
Bay of Bengal (India)	29.4 (−7.5)	5.37 (−358)	74.3 (−149)	40.4 (−168)	[75]
Kuwait Bay (Kuwait)	285.7 (−72.7)	31.0 (−2067)	17.5 (−35)	5.1 (−21.3)	[76]
Kakinada Bay (India)	130.3 (−33.2)	1.33 (−88.7)	28.6 (−57.2)	123.9 (−516)	[77]

^a^ Positive/negative values in red/green mean the times the results of this study are higher/lower compared to the others.

**Table 3:** Average values of total metal concentrations (mg/kg d.w.) in sediments from different aquatic ecosystems and their comparison with those from this study of the Algeciras Bay ^a^.

Site	Zn	Cd	Pb	Cu	Reference
Algeciras Bay	52.6	0.15	21.4	16.6	This study
Algeciras Bay	66.6 (−1.3)	0.48 (−3.2)	12.4 (+1.7)	18.4 (−1.1)	[93]
Cádiz Bay	101.8 (−1.9)	0.34 (−2.3)	17.3 (+2.3)	21.4 (−1.3)	
Huelva Estuary	1233.2 (−23.4)	10.0 (−66.7)	572 (−26.7)	2073.8 (−125)	[54]
Cádiz Bay	61.1 (−1.2)	0.13 (+1.2)	13.1 (+1.6)	51.0 (−3.1)	
Algeciras Bay	113.1 (−2.2)	-	9.4 (+2.3)	15.1 (+1.1)	[94]
Galician coast	158.0 (−3.0)	-	3.4 (+6.4)	2.2 (+7.5)	
Málaga Bay	-	0.076 (+2.0)	19.1(+1.1)	15.1 (+1.1)	[95]
Port of Maó	387 (−7.4)	0.07 (+2.1)	119 (−5.6)	79 (−4.8)	[96]
Bay of Biscay	20.9 (+2.5)	0.06 (+2.5)	6.3 (+3.4)	4.2 (+4.0)	[97]
Valencian coastline	30.8 (+1.7)	0.18 (−1.2)	6.0 (+3.6)	3.5 (+4.7)	[98]
Thessaloniki Bay (Greece)	218 (−4.1)	2.51 (−16.7)	84.2 (−3.9)	77.2 (−4.7)	[99]
Golfe-Juan Bay (France)	136.5 (−2.6)	0.27 (−1.8)	176 (−8.2)	49.4 (−3.0)	[100]
Edremit Bay (Turkey)	101 (−1.9)	0.15 (1.0)	20.7 (1.0)	26.5 (−1.6)	[101]
Todos Santos Bay (Mexico)	40.5 (+1.3)	-	11.9 (+1.8)	8.6 (+1.9)	[102]
Kompong Som Bay, Cambodia	47.3 (+1.1)	0.10 (+1.5)	23.7 (−1.1)	13.5 (+1.2)	[103]
Red Sea Coast, Jizan (Saudi Arabia)	24.7 (+2.1)	0.48 (−3.2)	3.9 (+5.5)	16.4 (1.0)	[73]
Qua Iboe Estuary (Nigeria)	121.6 (−2.3)	0.86 (−5.7)	5.2 (+4.1)	-	[74]

^a^ Positive/negative values in red/green mean the times the results of this study are higher/lower compared to the others.

## Data Availability

Data is contained within the article or Supplementary Materials.

## Supplementary Materials

The following supporting information can be downloaded at: https://www.mdpi.com/article/10.3390/toxics12030163/s1, Table S1: Geographical coordinates of sampling sites; Table S2: Analytical instruments and equipment; Table S3: Method detection limits of metal analysis; Table S4: BCR sequential extraction procedure and residue digestion for sediment fractionation; Table S5: Recoveries of the CRMs used for the assessment of the methodology accuracy; Table S6: Comparison of log K_d_ values with other estuaries and bays.
