# Supplementary material for: Ecological Status of Algeciras Bay, in a Highly Anthropised Area in South-West Europe, through Metal Assessment—Part I: Abiotic Samples
Source: Toxics. 2024 Feb 20;12(3):163. doi: 10.3390/toxics12030163 (PMC10974433; doi:10.3390/toxics12030163)
Supplement: Supplementary file 1 [file toxics-12-00163-s001.zip › toxics-2814850-supplementary.pdf]

Supplementary Material

Ecological Status of Algeciras Bay, in a Highly Anthropised Area in South-West Europe, through Metal Assessment—Part I: Abiotic Samples

María José Casanueva-Marenco, María Dolores Galindo-Riaño *, María Dolores Granado-Castro and Margarita Díaz-de-Alba

Department of Analytical Chemistry, Institute of Biomolecules (INBIO), Faculty of Sciences, International Campus of Excellence of the Sea (CEI-MAR), University of Cadiz, Campus Rio San Pedro, 11510 Puerto Real, Cadiz, Spain; mariajose.casanueva@uca.es (M.J.C.-M.); dolores.granado@uca.es (M.D.G.-C.); margarita.diaz@uca.es (M.D.-d.-A.)

* Correspondence: dolores.galindo@uca.es; Tel.: +34-956016362

Table S1. Geographical coordinates of sampling sites in Algeciras Bay

Sampling sites	Coordinates	
	**North**	**West**
1 – Getares beach	36° 05´ 28.31´´	5° 26´ 10.93´´
2 – Isla Verde	36° 07´ 8.43´´	5° 25´ 37.60´´
3 – Palmones	36° 10´ 19.51´´	5° 25´ 27.14´´
4 – Guadarranque	36° 10´ 32.21´´	5° 24´ 27.47´´
5 – Puente Mayorga	36° 10´32.23´´	5° 23´ 23.24´´

Table S2. Analytical instruments and equipment used in this study

Instrument and equipment	Specifications
Ultrapure water system	Millipore Milli-Q50 (Millipore, Burlington, Massachusetts, USA) (resistivity of 18.2 MΩ·cm at 25 °C)
Peristaltic pump	Masterflex 07571-05, popped head 07518-02, Cole-Parmer Instrument Co., (Vernon Hills, Illinois, USA)
Rigid Teflon pipes and flexible tubing	(Tygon, Masterflex, 6406-66).
Groundwater filter capsule	0.45 µm, Whatman (29705-92, Cole-Parmer Instrument Co., Vernon Hills, Illinois, USA)
Closed Teflon reactor	(PTFE, 100 mL, BRAND, 1305 38, Wertheim, Germany)
Electrochemical portable device	Model Sension 156 (Hach Co., Loveland, Colorado, USA)
Grab sampler	Model Van Veen grab (KC Denmark, Silkeborg, Denmark)
Vertical laminar flow cabinet (for samples and solutions)	Cruisair 870-FL (Cruma, Saint Boi de Llobregat, Barcelona, Spain)
Fume chamber (for acids and other fuming reagents)	Waldner 1800 (Wangen im Allgäu, Germany)
pH-meter	Basic 20 pH-meter with a 50_10T combined glass-Ag/AgCl electrode (Crison, Barcelona, Spain)
TOC analyser	Model 5050 (Shimadzu, Columbia, Maryland, USA)
Nylon filters	0.45 µm (Osmonics, Minnesota, USA)
Muffle furnace	Model N 20/Hr (Nabertherm, Lilienthal, Germany)
UV digester	Model 705 (Metrohm, Switzerland)
Shaker	Promax 2020 (Heidolph, Germany)
Centrifuge	4K10 Sigma (Osterode, Germany)
Hot water bath	20 L Precisterm (Selecta, Barcelona, Spain)
Infrared lamps	Siccatherm -SICCA 250 W, 240 V (Osram, Valencia, Spain)
Microwave assisted digester	Ethos 1600 (Milestone, Sorisole, Italy)
Differential Pulse Anodic Stripping Voltameter	746 VA Trace Analyzer processor with a Metrohm 747 VA Electrode Stand (Metrohm, Herisau, Switzerland) [a]
Inductively coupled plasma-mass spectrometer	X-Series ICP-MS equipment (Thermo Elemental, Winsford, UK) [b]

[a] An automated hanging mercury drop electrode as working electrode, an Ag/AgCl reference electrode (saturated with 3 mol/L KCl) and a platinum wire as auxiliary electrode were used.

[b] ICP-MS equipment was calibrated using ^{71}Ga, ^{103}Rh and ^{209}Bi as internal standards in order to minimise matrix interference effects.

Table S3. Method detection limits (MDL) (mg/kg) for the analysis of the different samples (n = 10)

Sample / method		Zn	Cd	Pb	Cu
Water / (DPASV)		0.66	0.01	0.04	0.25
Sediment: Total content / (ICP-MS)		0.18	0.008	0.03	0.07
Sediment: BCR fractionation / (ICP-MS)	F1	0.02	0.002	0.02	0.01
	F2	0.08	0.008	0.01	0.03
	F3	0.08	0.004	0.01	0.03
	F4	0.14	0.008	0.02	0.04

Table S4. The 3-step BCR sequential extraction procedure and residue digestion used for sediment fractionation

Step	Procedure
1	1 g of sediment sample was mechanically shaken with 40 mL of 0.11 mol/L acetic acid (CH_3COOH) for 16 h at 150 rpm in a Teflon vessel. Separation by centrifugation obtaining the extractable fraction.
2	Residue from Step 1 was shaken with 40 mL of 0.5 mol/L hydroxylammonium chloride ($NH_2OH{\cdot}HCl$) (adjusted at pH 1.5 by addition of HNO_3) for 16 h at 150 rpm. Separation by centrifugation obtaining the reducible fraction.
3	(i) Residue was twice immersed in a water bath at 85 ºC with 10 mL of 8.8 mol/L hydrogen peroxide (H_2O_2).<br>(ii) Extraction using 50 mL of 1 mol/L ammonium acetate (CH_3COONH_4) adjusted at pH 2 with HNO_3. Separation by centrifugation obtaining the oxidisable fraction.
Residue digestion	(i) Residual fraction was heated in a Teflon dish by using an IR lamp with 5 mL HF 48% (twice) and then 5 mL HNO_3 65% (twice), until complete dryness.<br>(ii) Residue was leached by magnetic shaking and heating for 1 h with 20 mL of 3.86 mol/L HCl. This extract was made up to a final volume of 25 mL.
Total acid digestion	The procedure of residue digestion was also carried out for the total acid digestion of sediments, but using 0.2 g of sediment and a final volume of 50 mL.

Table S5. Recoveries (%) of the CRMs used for the assessment of the accuracy of the methodology (n = 4)

**CRM**		**Recovery (%)**			
		**Zn**	**Cd**	**Pb**	**Cu**
BCR- 505		92.2 ± 5.1	88.8 ± 1.8	No certified *	107 ± 2
SRM 1646a		83.3 ± 2.6	101 ± 1	94.1 ± 10.1	85.3 ± 0.1
BCR- 701	F1	94.7 ± 0.5	101 ± 5	89.5 ± 10.3	117 ± 9
	F2	93.8 ± 4.2	96 ± 2	94.0 ± 6.2	73.8 ± 2.1
	F3	104 ± 11	110 ± 9	94.9 ± 6.4	101 ± 12

* Without certified value for Pb; indicative value given: 0.05 ± 0.03; found: 0.09 ± 0.01 µg/L

Table S6. Comparison of log K_d (L/kg) values for Zn, Cd, Pb and Cu in Algeciras with other estuaries and bays

Site	Zn	Cd	Pb	Cu	Reference
Algeciras Bay (Spain)	3.80−5.89	3.19−3.91	4.26−5.53	3.73−4.73	This study
Port Jackson Estuary (Australia)	4.7−5.2	-	-	3.9−5.1	[51]
Changjiang Estuary (China)	4.54−5.43	3.19−4.85	4.42−5.40	4.17−5.09	[52]
Yangtze Estuary (China)	-	3.7−3.9	-	4.6−4.8	[53]
Huelva Estuary (Spain)	3.23−4.57	3.15−4.73	3.98−5.09	4.15−5.04	[54]
Cadiz Bay (Spain)	2.3−4.5	3.3−4.6	2.6−5.2	3.5−4.9	
Sagami Bay (Japan)	-	4.2−5.9	-	3.8−6.2	[46]
Dakar coast and Saint Louis Estuary (Africa)	3.21	3.78	4.17	4.10	[55]
Bahía Blanca Estuary (Argentina)	-	2.2−5.1	-	3.8−6.0	[56]
El-Mex Bay (Egypt)	3.91−5.45	N.D.−6.02	N.D.−6.81	3.14−5.53	[57]
Zhanjiang Bay (China)	3.81−4.61	4.95−5.79	5.02−5.95	3.49−4.51	[47]
Masan Bay (Korea)	5.18 ± 0.52	4.37 ± 0.33	5.93 ± 0.48	4.47 ± 0.32	[58]
Loire Estuary (France)	4.9−5.7	3.5−5.0	-	4.1−5.0	[59]
